# *Thismia kelabitiana* (Thismiaceae), a new unique Fairy Lantern from Borneo potentially threatened by commercial logging

**DOI:** 10.1371/journal.pone.0203443

**Published:** 2018-10-03

**Authors:** Martin Dančák, Michal Hroneš, Michal Sochor, Zuzana Sochorová

**Affiliations:** 1 Department of Ecology and Environmental Sciences, Palacký University, Olomouc, Czech Republic; 2 Department of Botany, Palacký University, Olomouc, Czech Republic; 3 Department of Genetic Resources for Vegetables, Medicinal and Special Plants, Centre of the Region Haná for Biotechnological and Agricultural Research, Crop Research Institute, Olomouc, Czech Republic; Nanjing Agricultural University, CHINA

## Abstract

*Thismia kelabitiana*, a new unique species from the Sarawak state of Malaysia in the island of Borneo is described and illustrated. This new species is not similar to any species of *Thismia* described so far especially by having a unique form of mitre and outer perianth lobes deeply divided into 8–10 acute lobes and forming striking fringe around perianth tube opening. The species appears to be critically endangered due to ongoing logging activities in the region. It may potentially become a surrogate species for lower montane forests of the region and thus help protect them against further destruction.

## Introduction

Mycoheterotrophs, i.e. achlorophyllous plants able to obtain carbon via the mycorrhizal fungi associated with their roots, are important components of primary tropical rain forest biodiversity across the globe [1]. The island of Borneo is still covered with significant primary forests, although their area has been rapidly decreasing in the past decades [2]. Commercial logging and subsequent land conversion (mostly for oil palm plantations) drastically reduced the area of pristine old-growth forest from 55.8 Mha in 1973 to 20.6 Mha in 2015 [3].

Borneo’s rainforests host enormous diversity of mycoheterotrophic plants: twenty genera belong to seven families with at least 70 described species, i.e. 17% of the global biodiversity of mycoheterotrophs, orchids with 13 genera and more than 30 species being the richest family. Most of them have been poorly studied, and the knowledge on their taxonomy (as well as ecology and distribution) has been increasing only in recent years, e.g. [4], [5], [6], [7], [8].

An excellent example of this group may be *Thismia*, a genus of more than 75 species of mycoheterotrophic plants distributed mainly in tropical regions of Asia and the Americas, Borneo with 19 species [9], [10] being its centre of diversity. *Thismia* species usually prefer primary tropical rainforests–a habitat that faces unprecedented destruction in Borneo. Nature conservation efforts in this region could be potentially supported by discoveries of charismatic organisms that easily attract attention of the public, e.g. [11]. Such species may become surrogate species, i.e. species that are used to represent other species or aspects of the environment to attain a conservation objective [12]. Surrogate species are widely used in nature conservation as a shortcut for conservation planning [13] especially in regions where detailed data on biodiversity are lacking [14]. Umbrella and flagship species are generally known examples of such surrogate species. They are usually large mammals such as apes, pachyderms and carnivores or other distinctive animals as birds of prey and butterflies [15] that attract attention of the general public. Among plants, notable examples are orchids and parasitic *Rafflesia* [16] famous thanks to its large flowers which appear in tropical forests of Asia. Although *Thismia* species are tiny and usually inconspicuous plants in comparison to large mammals or *Rafflesia*, in close view they are fairly strange-looking and some of them are attractive and colourful. Their odd appearance, together with their peculiar life strategy and strong links to the primary forests, makes them exceptional among rainforest herbs. Thus, they are potential candidates for good surrogate species.

A few years ago, we received photographs of an unknown achlorophyllous plant, which was accidentally discovered by a group of our colleagues on a hike to the Kelabit Highlands of Sarawak in 2010. The pictures showed an undoubtedly undescribed striking species of *Thismia*. In 2017, we therefore launched an expedition to find the locality and the plant from these photographs. The successfully discovered plant with a series of unique traits is described here as a new species. Owing to its size, colour and distinctive morphology, this species is among the most eye-catching representatives of the genus.

## Materials and methods

### Ethic statement

The new species reported in this study was collected from a forest site located outside any protected area of Sarawak State of Malaysia. The research as well as collection and export of plant material was permitted and approved by relevant Sarawak authorities. Since this species is currently undescribed, it is inevitably not yet included in any of the existing Red Lists and lists of protected species.

### Morphological observations

Morphology of the new species was studied in a field camp using hand lenses (20×–60× magnification) and macrophotography. Specimens of whole plants were taken from the type subpopulation in form of pressed herbarium and alcohol (70% ethanol) specimen and deposited in SAR and OL.

### Nomenclature

The electronic version of this article in Portable Document Format (PDF) in a work with an ISSN or ISBN will represent a published work according to the International Code of Nomenclature for algae, fungi, and plants, and hence the new names contained in the electronic publication of a PLOS ONE article are effectively published under that Code from the electronic edition alone, so there is no longer any need to provide printed copies.

In addition, the new name contained in this work has been submitted to IPNI, from where it will be made available to the Global Names Index. The IPNI LSIDs can be resolved and the associated information viewed through any standard web browser by appending the LSID contained in this publication to the prefix http://ipni.org/. The online version of this work is archived and available from the following digital repositories: PubMed Central, LOCKSS.

## Results

### Taxonomic treatment

**Fig 1:**
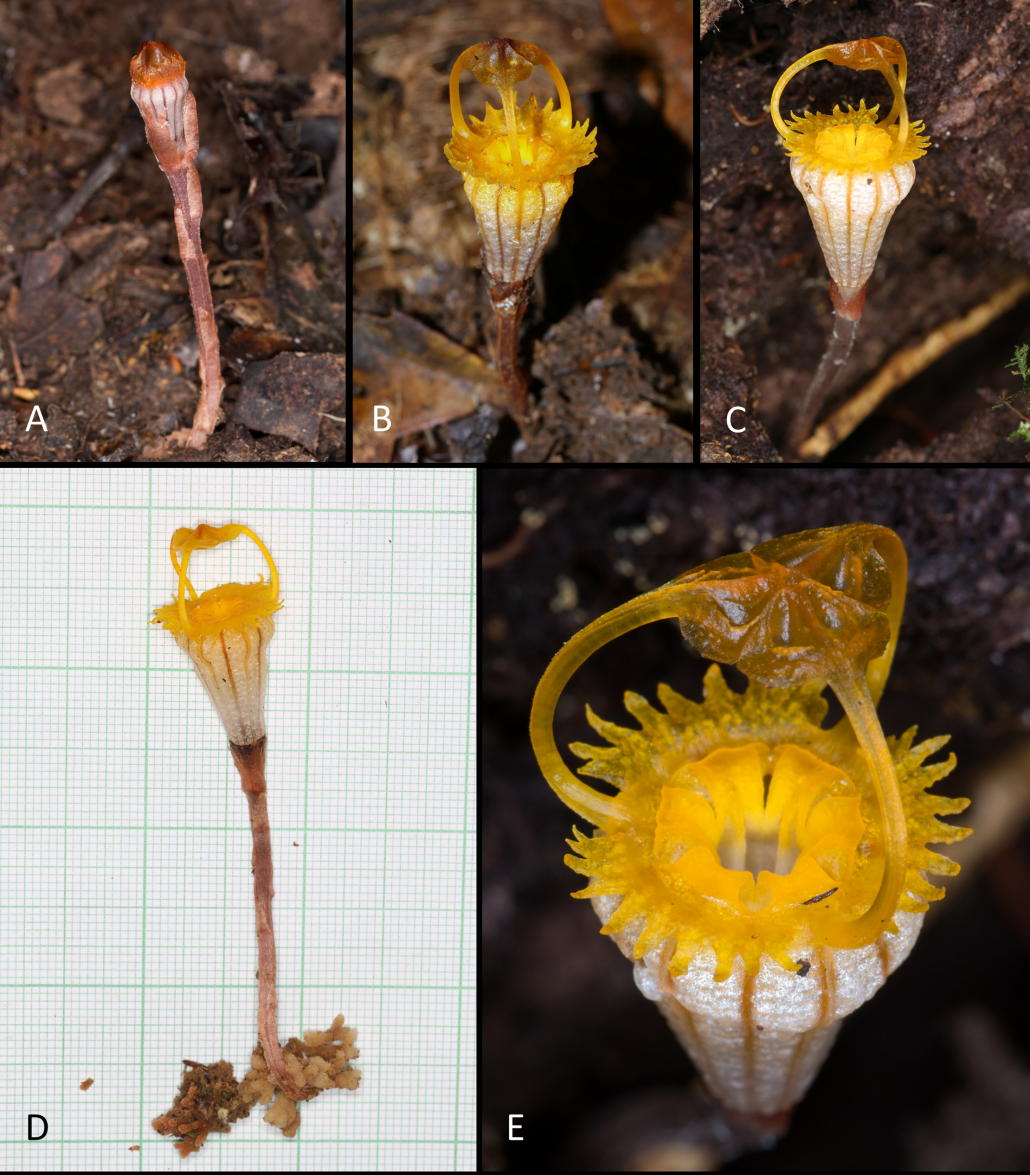
*Thismia kelabitiana*. **A**, Plant with flower bud. **B**, Plant with young flower. **C**, Plant with mature flower. **D**, Whole plant with root system. **E**, Detail of mitre and perianth opening. Photos Michal Sochor.

**Fig 2:**
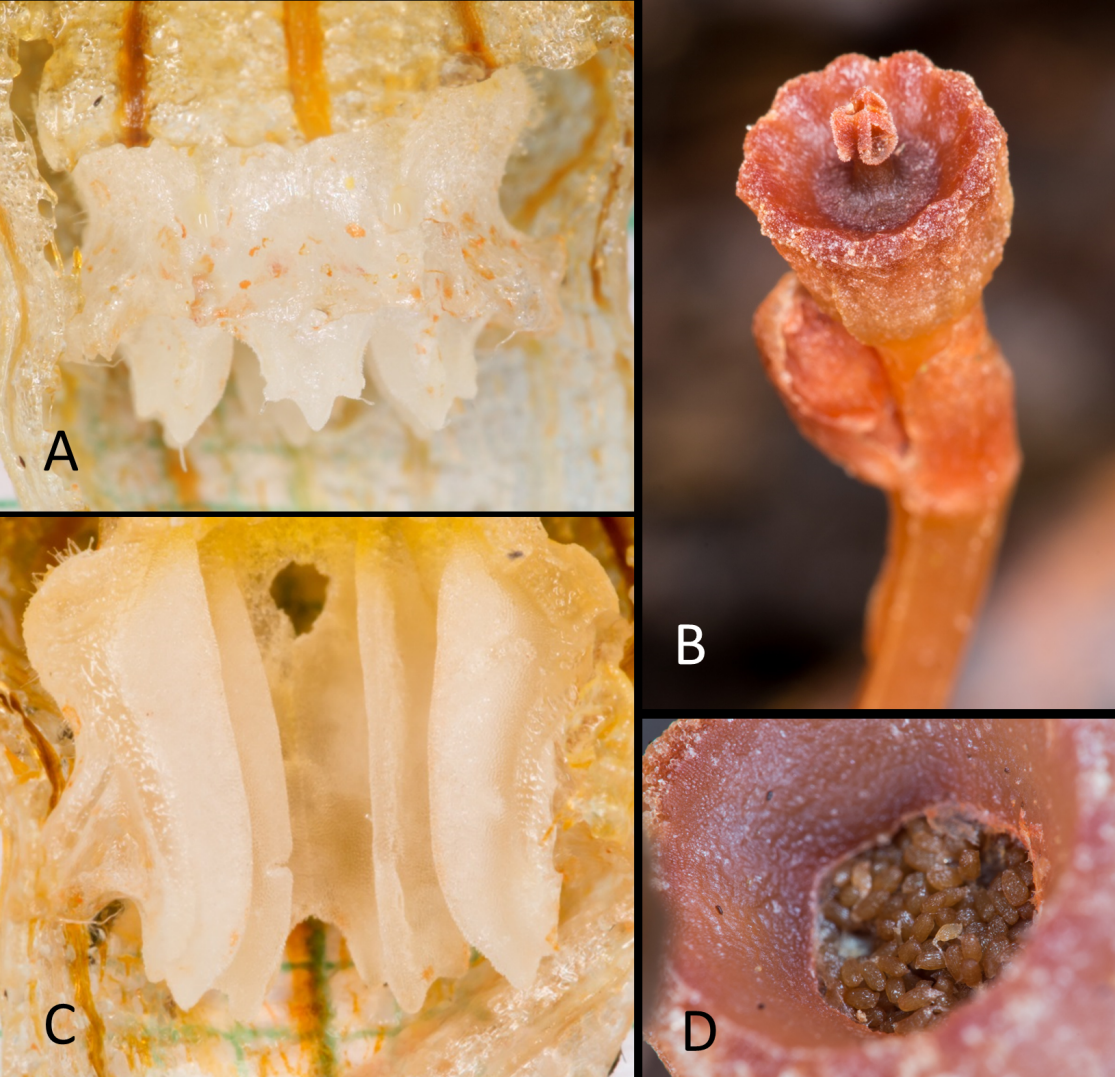
*Thismia kelabitiana*. **A**, Outer view of stamens showing the lateral appendages and apical parts of connectives. **B**, Young capsule with persistent stigma. **C**, Inner view of stamens showing ribbed connectives. **D**, Seeds inside mature capsule. Photos Michal Sochor.

**Fig 3:**
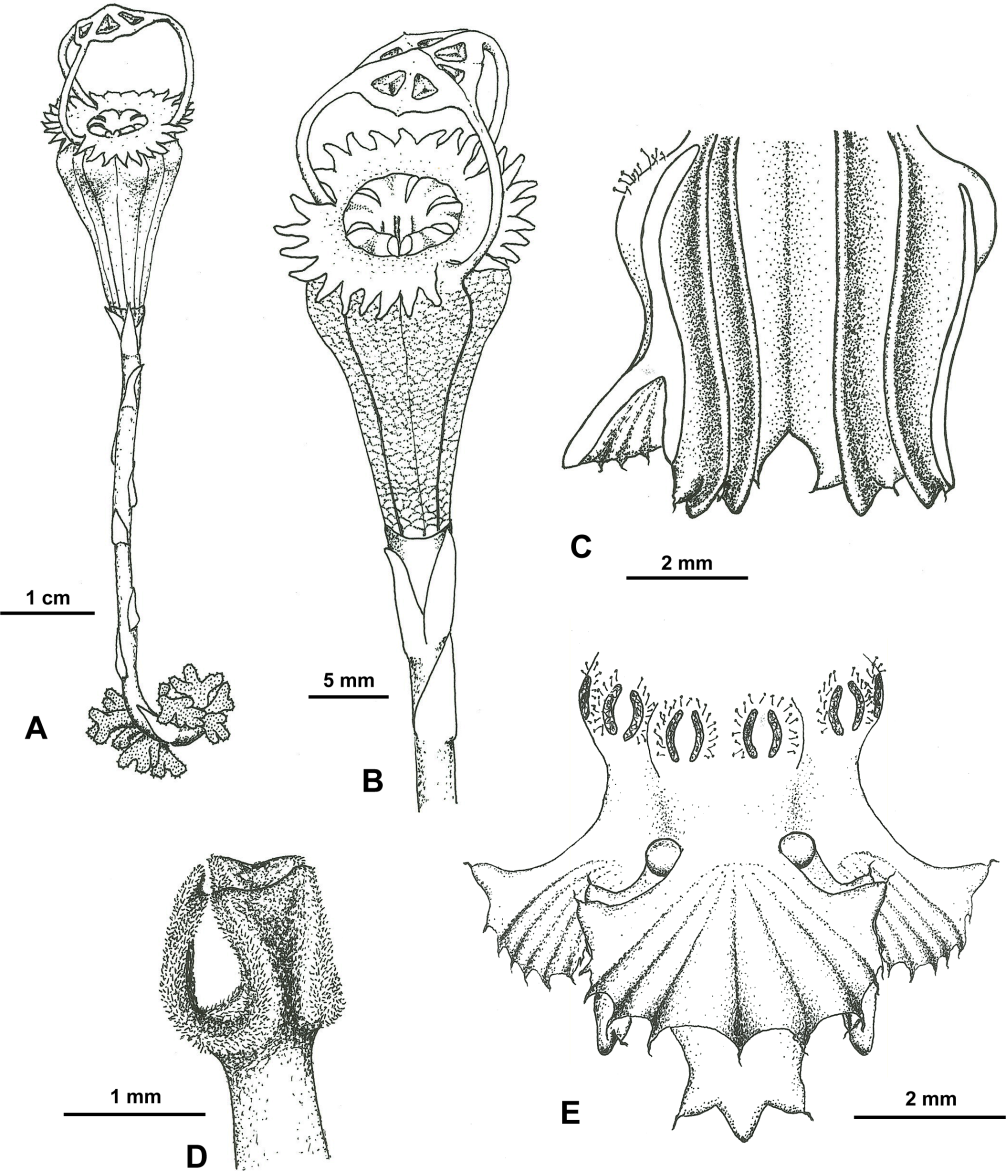
*Thismia kelabitiana*. **A**, Habit of flowering plant. **B**, Side view of flower. **C**, Inner view of stamens. **D**, Style with stigma. **E**, Outer view of stamens. Drawn by Kateřina Janošíková.

***Thismia kelabitiana*** Dančák, Hroneš & Sochor, *sp*. *nov*. [urn:lsid:ipni.org:names: 77187919–1] (Figs 1, 2 and 3).

**Type.** MALAYSIA, Sarawak: Kelabit Highlands, Pa'Umor village, Anak Kadi Ridge, 4.4 km SSE of the village. Coordinates WGS 84: N 03°42'; E 115°31', Elevation 1195 m a.s.l., 13 January 2017. M. Sochor, M. Hroneš, M. Dančák, Z. Egertová & J.R. Pasan BOR1/17 (holotype SAR [in spirit and herbarium specimen, accession number Sochor/BOR-1/17], isotype OL [35272]).

**Diagnosis.** *Thismia kelabitiana* differs significantly from all congeneric species by the combination of the following traits, e.g., flowers large (up to 2.8 × 1.8 cm), outer perianth lobes deeply divided into 8–10 acute lobes and forming striking fringe around perianth tube opening, mitre relatively small and flat elevated by three long filiform pillars, connectives with prominent longitudinal rib and three appendices on apical margin.

**Description.** Achlorophyllous herb, *ca*. 5–18 cm tall. Roots short, clustered, coralliform, light brown. Stem 1.5–16 cm long and 2 mm in diameter, erect (or sometimes ascending), simple or sparsely branched in upper part (branches developing after anthesis), longitudinally ribbed, dark pinkish to reddish brown (to almost grey or orange); bearing 1 or 2(–3) flowers. Leaves (3–)6–10, spirally arranged, scale-like, triangular, acute to acuminate, entire, 4.5–5.5 mm long and 1.8–2 mm wide at base, light brown to pinkish. Bracts 3, widely triangular to ovate, entire to irregularly dentate and often deeply torn, *ca*. 6–8 × 2.5–4 mm, pinkish to brown. Flowers sessile, actinomorphic, 2.6–2.8 cm long; perianth tube of 6 fused tepals, funnel-shaped in the basal part, urceolate at the apical part, widest (1.2–1.3 cm) at its upper quarter, white to bright yellow at the top, with six brownish non-prominent longitudinal ribs and six yellow to brown longitudinal stripes on outer surface, inner surface net-like structured; outer perianth lobes falcate in outline, much wider than long, *ca*. 9 mm wide and 5 mm long, deeply divided into 8–10 acute lobes, bright yellow, arranged in one plane and together forming striking fringe around mouth of perianth tube, 1.6–1.8 cm in diameter; inner perianth lobes bright yellow to brownish-yellow, turned upwards, connate at the end and forming mitre, the proximal part of the lobe filiform, bent upwards, pillar-like, *ca*. 1.2 cm long and 0.8–1.0 mm wide in diameter, the distal part of the lobe ± flat, rhombic in outline, with central rib and two tetrahedral depressions on upper surface of each lobe; the lobes joined to form almost flat mitre, roundly triangular in outline, 7–8 mm wide. Stamens 6, pendent from the apical margin of the perianth tube; annulus absent; filaments yellow, curved downwards, with bases slightly emerged above perianth tube apex, not connate and forming six apertures apparent from upper view; connectives broad, laterally connate to form a tube, *ca*. 7 mm long, each with prominent longitudinal rib extending along the whole length of the inner side of the connective, apex of each connective with one central lobe (extension of the rib) and two smaller lobes pointing somewhat centrifugally and bearing one transparent trichome each; lateral appendage box-shaped, protruding towards perianth tube, not reaching the apex of the connective, shallowly dentate and sparsely hairy on free margins; thecae whitish, surrounded by tufts of hairs; interstaminal glands inserted on the line of fusion between connectives. Style short, stigma 3-lobed, papillose, lobes ± rectangular, longitudinally furrowed; ovary inferior, obconical, dark brown-reddish, covered by bracts. Capsule cup-shaped, 5–7 mm in diameter, dark brown to blackish or reddish before maturity, later pinkish, on very short pedicel; seeds numerous, brown, ellipsoid, *ca*. 0.3 × 0.5 mm.

**Variability.** The species is rather uniform, although only *ca*. 20 individuals were seen altogether. Stem is rather variable in colour and its length varies from 1.5 cm to 16 cm, which may be attributed to phenotypic plasticity likewise in many other species of the genus. The number of flowers is usually two, a few three-flowered individuals were also observed. Almost no variability was detected in flower size, colour and structure.

**Fig 4:**
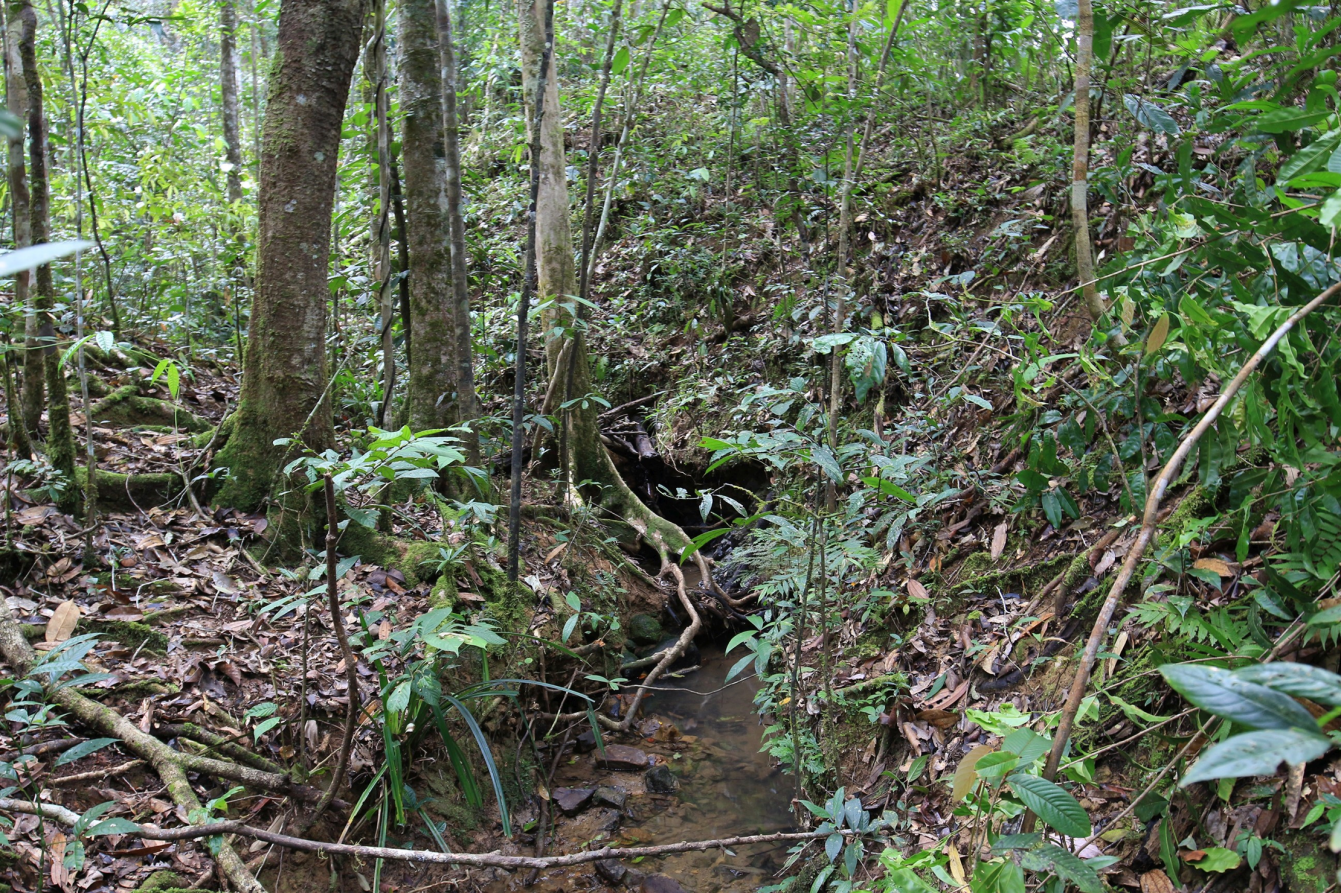
Habitat of *Thismia kelabitiana*. Ravine of a small stream in lower montane tropical rain forest. Photo Michal Sochor.

**Habitat and ecology.** The species occurs in lower montane primary tropical rainforest at an altitude around 1200 m a. s. l. It was found in humid stream ravines as well as in relatively drier open forest sites (Fig 4). A variety of other mycoheterotrophic species were abundant at the type locality, including *Aphyllorchis pallida*, *Burmannia lutescens* agg., *B*. *championii*, *Cystorchis aphylla*, *Gymnosiphon aphyllus* agg., *Epirixanthes kinabaluensis*, *Exacum tenue*, *Platanthera saprophytica*, *Sciaphila arfakiana*, *S*. cf. *nana*, *S*. *tenella*, *Thismia cornuta*, *T*. *minutissima* ined., *T*. aff. *nigra* and *T*. *viridistriata*. Herbaceous vegetation was otherwise sparse. *Thismia kelabitiana* seems not to prefer any particular environmental conditions at the locality as it occurs in various aspects of slopes with various inclinations either in rugged ravines or relatively flat terrain in various distances from a stream.

**Distribution.** The species is known only from the type locality and the other, 600 m distant locality, where plants have only been photographed. Both localities are found on a ridge south-east from Pa’Umor village in the Bario district of Sarawak (Malaysia).

**Etymology.** The specific epithet reflects the geographical origin of the species in the Kelabit Highlands, the land of the Kelabit people.

**Common name.** Pa’Umor Fairy Lantern. There is no widely accepted generic common name for the genus *Thismia*. The recent wave of interest as well as potential conservational employment, nevertheless, calls for this name. Sometimes the name Fairy Lantern is used [17], [18] which has originally belonged to *Thismia rodwayi* (either in singular or plural form as Fairy Lanterns). Since the name has already been used for a few other *Thismia* species, we follow this approach. We suggest calling *Thismia kelabitiana* Pa’Umor Fairy Lantern. The name is derived from Pa’Umor village community in whose forest the species occurs.

**Conservation status.** Both known subpopulations occur in primary rainforest that does not have any official protection status and falls under logging concessions [19]. Although the species may be locally common and the possibility of its occurrence in neighbouring regions of Sarawak and Indonesia cannot be excluded, its distribution is probably limited and scarce. It is one of the most robust *Thismia* species so far recorded, with conspicuous yellow flower and attractive appearance. Despite this, it was not recorded from nearby national parks Pulong Tau and Kayan Mentarang. The extent of occurrence (EOO) of *T*. *kelabitiana* cannot be estimated because the species is only known from the type collection. The sites of the two subpopulations represent one location (*sensu* IUCN [20]; a geographically or ecologically distinct area in which a single threatening event can rapidly affect all individuals of the taxon present) and its area of occupancy (AOO) is estimated to be 4 km^2^, which falls within the limits for Critically Endangered status under criterion B2 of IUCN Red List Categories and Criteria [20]. Moreover none of the subpopulations contains more than 50 mature individuals. The species therefore fulfils criteria CR B2ab (iii,iv,v) and C2a(i), as well.

## Discussion

### Taxonomic affinities

Having coralliform roots and three perianth lobes that are connate at the top to form a mitre, the species clearly belongs to *Thismia* sect. *Sarcosiphon* [21]. However, it is unique among the known *Sarcosiphon* species in having several unparalleled traits. The flowers are relatively large among *Thismia* species, with almost 3 cm in length and nearly 2 cm in width are among the most robust in the whole genus. The form of mitre is also unique as it is relatively small, flat and positioned high above the perianth opening elevated by three filiform pillars. Furthermore, the presence of striking fringe around the perianth opening, apparently representing the outer perianth lobes, is very distinctive. Ribbed connectives are rare in the whole genus as well. Undoubtedly the most similar of all species of *Thismia* is *T*. *goodii* from Sabah [22]. In this species the outer perianth lobes are also extremely wide and short (although they are entire on margin) thus they form a discontinuous ring around the opening of the perianth tube and its mitre is structurally similar to that of *T*. *kelabitiana* as well. Although the drawing of connectives is not so detailed in the protologue of *T*. *goodii*, it is clear from the description that they are likewise very similar to those of *T*. *kelabitiana*. Interestingly, *Thismia clavigera* has extremely similar inner structure of the flower, though it was once classified in family monographs [23], [24] to another genus *Geomitra* on the basis of three long appendages arising from the top of the mitre. The only other known member of the former *Geomitra*, *Thismia betung-kerihunensis* shows some similarities to *T*. *goodii* as well (and hence also to *T*. *kelabitiana*), especially in the form of mitre. However, the rather poor description of connectives in the protologue of *T*. *betung-kerihunensis* [25] does not allow for comparison with the other species. These four species therefore might be in fact closely related and the presence of mitre appendages could be variable among them. Another pair of *Thismia* species, *T*. *gigantea* and *T*. *appendiculata*, i.e. genus *Scaphiophora* sensu Jonker, also shows some similarities to *T*. *kelabitiana*, especially in the form of the mitre. Nevertheless, they have a single erect appendage arising from the apex of the mitre. This pair of species belongs, however, among the least known species of *Thismia*, both being collected only once in the Philippines and New Guinea, respectively [23].

### Conservational implications

We believe that *T*. *kelabitiana* may well become a surrogate species for lower montane primary tropical rainforest of the Bario part of the Kelabit Highlands. Thanks to its distinctive look, it is readily recognizable and thus may serve as a flagship species. As the only effective way of its protection is the conservation of its habitat, it is also a good candidate for an umbrella species. Forests in the southern part of the Kelabit Highlands have been almost completely disturbed by a dense network of logging and connection roads and subsequent selective logging. The nearest, newly-built road is less than 3 km from the type locality. However, the relatively small area (dozens of km^2^) of primary forests between Pa’Umor village and abandoned village of Pa’Main remains intact and the local community makes a remarkable effort to protect their land despite the fact that it is still under the logging concession. One possible way of helping to save at least some aspects of the traditional way of life of the Kelabit people that does not hamper socioeconomic development is ecotourism. Besides orchids and carnivorous plants, which traditionally attract public attention, *Thismia kelabitiana* may be another plant species that will increase the attractiveness of the region for ecotourism. As a flagship species, *T*. *kelabitiana* may then help local initiatives in obtaining any official protection status for the Pa’Umor forests, from Community Forest to a National Park.
